# Supplementary material for: Structure of the human Parkin ligase domain in an autoinhibited state
Source: EMBO J. 2013 May 31;32(15):2099–112. doi: 10.1038/emboj.2013.125 (PMC3730226; doi:10.1038/emboj.2013.125)
Supplement: Supplementary Information [file emboj2013125s1.pdf]

Structure of the human Parkin ligase domain in an autoinhibited state

Supplementary Material

Tobias Wauer and David Komander[1]

Medical Research Council Laboratory of Molecular Biology, Cambridge, UK.

[1] Corresponding author: David Komander, dk@mrc-lmb.cam.ac.uk

Running title: Structure of the Parkin UPD-RBR

Contents:

3 Supplementary Figures
1 Supplementary Table
Supplementary References

Supplementary Figure 1

A Experimental electron density map, phased by Zn-SAD

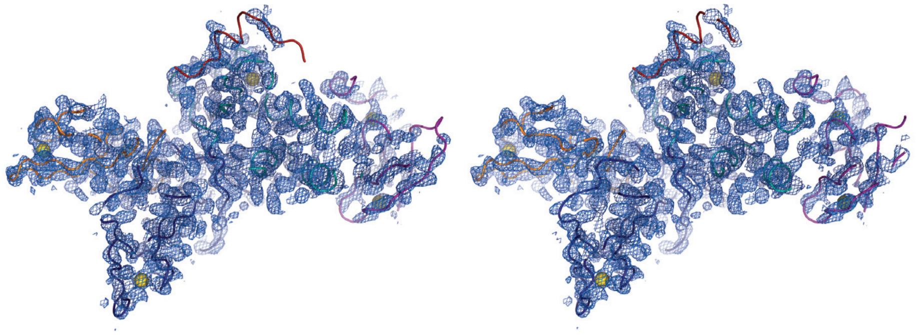

B Final refined electron density map

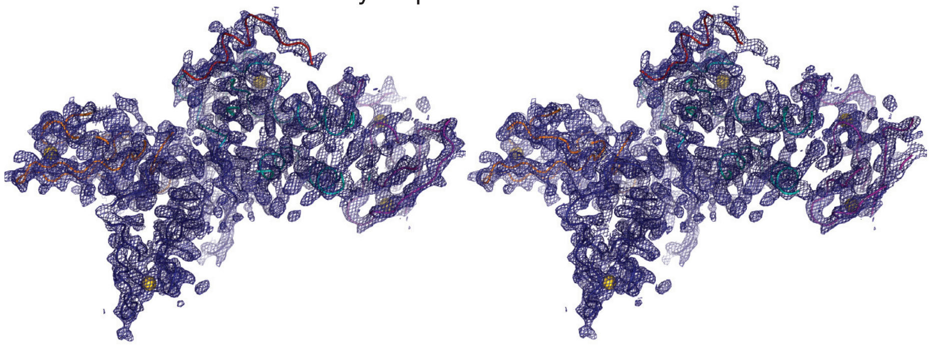

C RING2 positioning in crystal structures

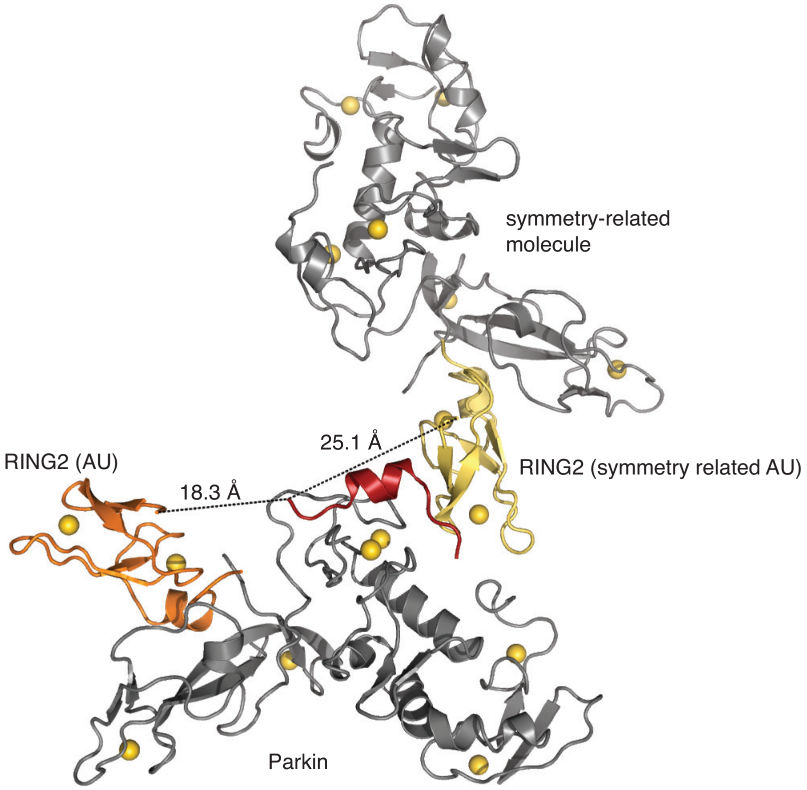

Supplementary Figure 1. Electron density maps

Stereo picture of electron density covering Parkin in the asymmetric unit, shown as a ribbon diagram coloured as in **Figure 1B**. Zn atoms are shown as yellow spheres. **(A)** Experimental electron density map, contoured at 1 σ, after density modification by SHELXE. **(B)** Refined 2|Fo|-|Fc| electron density map contoured at 1 σ. **(C)** Subdomain connectivity was initially ambiguous, as the disordered region between the linker helix and RING2 (aa 405-412) could reach RING2 of two neighbouring asymmetric units (AUs). The shorter distance (18.3 Å) connects the linker to the RING2 in the AU, as this domain forms strong hydrophobic contacts with the UPD, and RING2 would be autoinhibited. The longer distance (25.1 Å) would connect to a RING2 domain that would expose its catalytic Cys, and contact RING1, IBR and the linker helix, albeit only with few polar contacts. Our mutational and biochemical analysis makes the latter interface more unlikely. It was a possibility that the alternative RING2 conformation resembled that of an active 'open' state of Parkin (compare **Figure 5B**), but modelling of the E2~Ub (as in **Figure 5A**) led to significant clashes of the E2 with RING2 in the alternative position.

Supplementary Figure 2

A

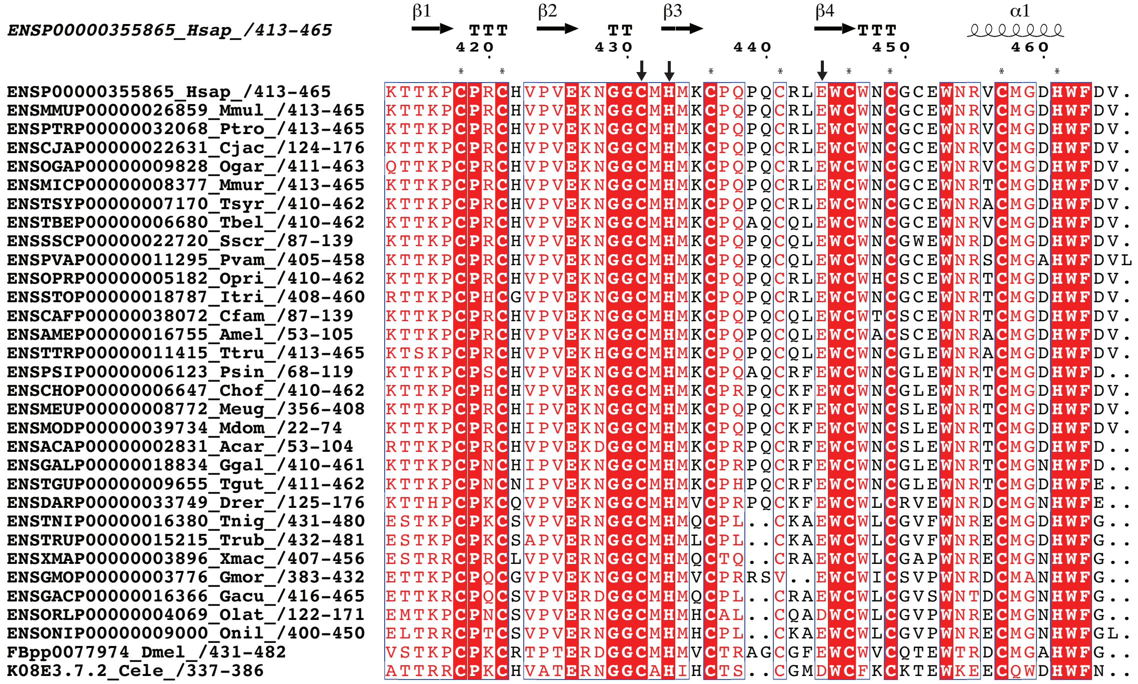

B

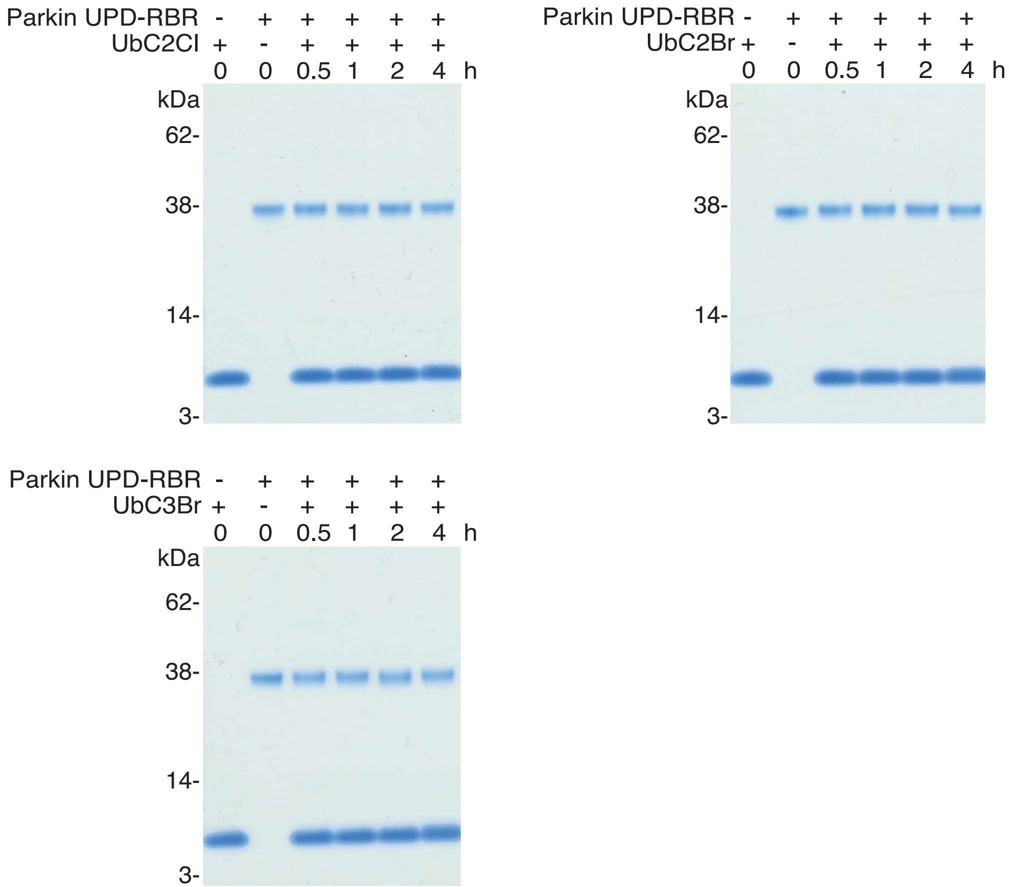

Supplementary Figure 2. RING2 conservation in Parkin

Species alignment based on Parkin sequences annotated in ensemble (http://www.ensembl.org/Homo_sapiens/Gene/Sequence?g=ENSG00000185345;r=6:161768452-163148803) that contain a fully annotated RING2 sequence. Secondary structure and numbering based on the human sequence and according to the crystal structure is indicated above the alignment. **(B)** Ub-based suicide inhibitors are unable to modify the crystallised Parkin UPD-RBR fragment. Coomassie-stained SDS-PAGE gels are shown for incubation of Parking with Ub-based suicide probes for indicated amount of time. The used Ub-haloalkyl probes are described in (Borodovsky *et al*, 2002).

Supplementary Figure 3

A

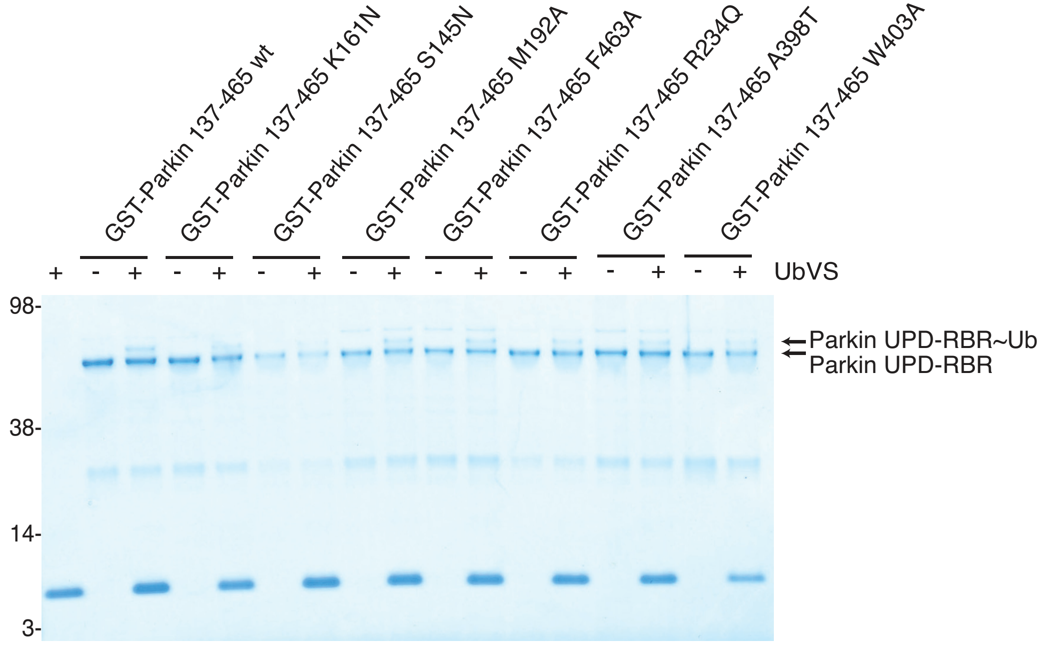

B

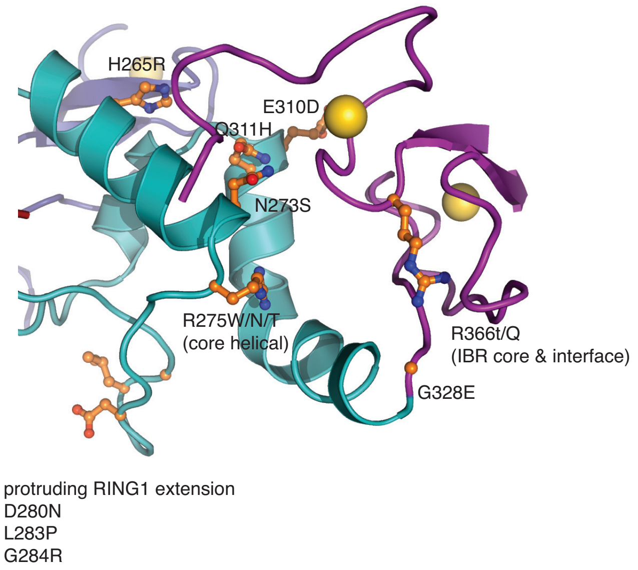

C

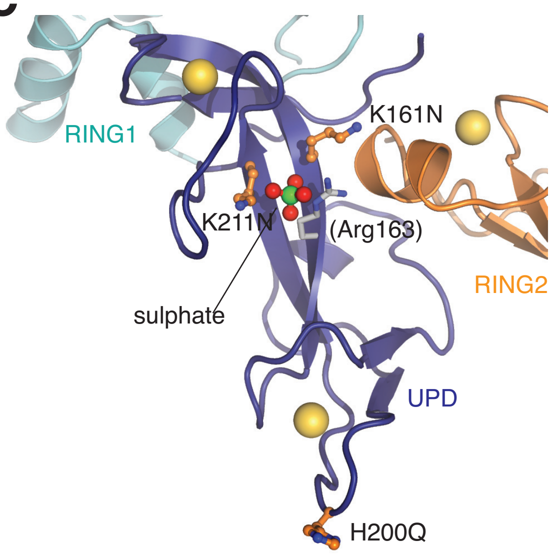

Supplementary Figure 3. Structural detail of Parkin mutations

(A) modification of indicated GST-tagged Parkin UBP-RBR variants with Ub-vinylsulfone (UbVS). The GST-tag on wt UPD-RBR fragment activated the protein, which is modified with Ub-VS, and an increase in reactivity cannot be observed in this assay. **(B)** Parkin mutations in the RING1:IBR domain interface, including two core residues mutation of which may change the structure. Also, the three surface exposed mutations in the unique protruding β-hairpin of the RING1 domain are shown. This mutations hotspot indicates functional relevance, most likely in interactions with other proteins. **(C)** View of the UPD in a ~180º rotated orientation as in **Figure 1B** (note RING2 on right hand side) showing three patient mutations. His200 is fully exposed. The sulphate ion is shown in ball-and-stick representation with a green sulphur atom and red oxygen atoms.

Supplementary Table I. Known Parkin mutations in the crystallized construct

Mutation	Domain	Predicted structural rationale	Biochemical effect
Zn-coordinating residues			
**C166Y**	UPD	Coordinates UPD Zn2	
**C212G**<br>**C212Y**	UPD	Coordinates UPD Zn1	Reduced solubility (Wang *et al*, 2005)
**H215Q**	UPD	Coordinates UPD Zn1	
**C238Q**	RING1	Coordinates RING1 Zn1	
**C253Y**<br>**C253W**	RING1	Coordinates RING1 Zn2	
**R256C**	RING1	Creates a competing Cys for RING1 Zn2 binding	- Reduced complex formation (Van Humbeeck *et al*, 2008)<br>- Reduced solubility (Wang *et al*, 2005)<br>- Autoubiquitination, but reduced substrate ubiquitination (Sriram *et al*, 2005)
**H257R**	RING1	Coordinates RING1 Zn2	
**C289G**	RING1	Coordinates RING1 Zn2	- Reduced solubility (Wang *et al*, 2005; Gu *et al*, 2003)
**R334C**	IBR	Creates a competing Cys for IBR Zn1 binding	- Reduced solubility (Wang *et al*, 2005)
**R402C**<br>**R402H**	Linker helix	May interfere with Zn1 binding of RING1	
**C418R**	RING2	Coordinates RING2 Zn1	- Reduced solubility (Gu *et al*, 2003)
**C441R**	RING2	Coordinates RING2 Zn1	- Reduced solubility (Wang *et al*, 2005; Hampe, 2006)
Domain core residues			
**P153R**	UPD	In UPD Zn1 binding loop	
**T173M**	UPD	In UPD Zn2 binding loop	
**T240R**<br>**T240M**	RING1	In RING1 Zn1 binding loop<br>May affect E2 binding	- Ligase dead (Sriram *et al*, 2005)<br>- Reduced mitochondrial localization (Matsuda *et al*, 2010)
**V258M**	RING1	RING1 core residue, may affect linker helix binding	
**Y267H**	RING1 helical	Core residue of RING1 helical domain	
**C268R**	RING1 (helical)	Core residue of RING1 helical domain	
**R275N**<br>**R275W**<br>**R275T**	RING1 (helical)	RING1 helical domain core residue	- Reduced solubility (Wang *et al*, 2005)<br>- Increased ubiquitination activity (Sriram *et al*, 2005)<br>- No mitophagy (Narendra *et al*, 2010; Geisler *et al*, 2010)
**I298S**<br>**I298L**	RING1	RING1 core residue	

**T351P**	IBR	Disrupt IBR β-sheet, next to Zn-coordinating Cys	IBR domain destabilisation (Beasley *et al*, 2007)
**R366T**<br>**R366Q**	IBR	IBR core residue, also on interface with RING1	
**P437L**<br>**P437I**	RING2	Near Zn1 binding site	Ligase dead (Sriram *et al*, 2005)
<u>Residues affecting ubiquitination mechanism</u>			
**D243N**	RING1	Forms contact with conserved Lys on E2 helix 1	
**C431F**	RING2	Active site Cys	- Reduced solubility (Wang *et al*, 2005; Sriram *et al*, 2005)<br>- Increases autoubiquitination (Sriram *et al*, 2005)<br>- No substrate ubiquitination (Sriram *et al*, 2005)<br>- Reduced translocation to mitochondria (Lazarou *et al*, 2013)
**E444Q**	RING2	Potential catalytic triad residue (unconfirmed)	
<u>Interface residues between RING2 & UPD</u>			
**S145N**	UPD	Destabilises interface?	
**M192L**<br>**M192V**	UPD	Destabilises interface?	
**S193I**	UPD	Destabilises interface?	
**G429D**	RING2	Destabilises interface?	
**G430D**	RING2	Destabilises interface?	- Ligase dead (Chew *et al*, 2011)<br>- Increased auto-ubiquitination (Sriram *et al*, 2005)<br>- Reduced substrate ubiquitination (Sriram *et al*, 2005)
**M458L**	RING2	Near RING2 UPD interface but exposed	
**A230T**	RING1	May create RING1/RING2 interface.<br>May affect Linker helix binding<br>May affect RING1 structure	
<u>Interface residues between RING1 and linker helix</u>			
**R234Q**	RING1	May affect linker helix binding	
**A398T**	Linker helix	Disrupts linker helix binding	
**A401D**	Linker helix	Disrupts linker helix binding	
<u>Residues in RING1:IBR interface</u>			
**N273S**	RING1	Destabilises interface?	
**E310D**	RING1	Destabilises interface?	

**Q311H**	RING1	Destabilises interface?	
**G328E**	RING1	Loop between RING1 and IBR<br>Destabilises interface?	- Increased auto-ubiquitination (Sriram *et al*, 2005)<br>- Reduced substrate ubiquitination (Sriram *et al*, 2005)<br>- Reduced solubility (Wang *et al*, 2005)
Putative phospho-peptide binding site			
**K161N**	UPD	- Phospho-peptide binding site?<br>- Salt bridge to Ring2	- Reduced complex formation (Van Humbeeck *et al*, 2008)<br>- Ligase dead (Sriram *et al*, 2005)<br>- Ubiquitinates substrate (Hampe *et al*, 2006)<br>- Reduced mitochondria clearance (Geisler *et al*, 2010)
**K211N**	UPD	Phospho-peptide binding site?	- Reduced complex formation (Van Humbeeck *et al*, 2008)<br>- Reduced mitochondrial localization (Matsuda *et al*, 2010)
Residues with unclear effects on Parkin function			
**H200Q**	UPD	Exposed in UPD	
**V244I**	RING1	On RING1, exposed	
**H265R**	RING1 helical	On RING1, exposed	
**D280N**	RING1	β-sheet insertion in RING1	Reduced solubility (Wang *et al*, 2005)
**L283P**	RING1	β-sheet insertion in RING1	
**G284R**	RING1	β-sheet insertion in RING1	
**T415N**	RING2	Exposed in RING2.	Ligase dead (Sriram *et al*, 2005; Matsuda *et al*, 2010)

Supplementary References

Beasley SA, Hristova VA & Shaw GS (2007) Structure of the Parkin in-between-ring domain provides insights for E3-ligase dysfunction in autosomal recessive Parkinson's disease. *Proc Natl Acad Sci USA* **104:** 3095–3100

Borodovsky A, Ovaa H, Kolli N, Gan-Erdene T, Wilkinson KD, Ploegh HL & Kessler BM (2002) Chemistry-based functional proteomics reveals novel members of the deubiquitinating enzyme family. *Chem Biol* **9:** 1149–1159

Chew KCM, Matsuda N, Saisho K, Lim GGY, Chai C, Tan H-M, Tanaka K & Lim KL (2011) Parkin mediates apparent E2-independent monoubiquitination in vitro and contains an intrinsic activity that catalyzes polyubiquitination. *PLoS ONE* **6:** e19720

Geisler S, Holmström KM, Skujat D, Fiesel FC, Rothfuss OC, Kahle PJ & Springer W (2010) PINK1/Parkin-mediated mitophagy is dependent on VDAC1 and p62/SQSTM1. *Nat Cell Biol* **12:** 119–131

Gu W-J, Corti O, Araujo F, Hampe C, Jacquier S, Lücking CB, Abbas N, Duyckaerts C, Rooney T, Pradier L, Ruberg M & Brice A (2003) The C289G and C418R missense mutations cause rapid sequestration of human Parkin into insoluble aggregates. *Neurobiol. Dis.* **14:** 357–364

Hampe C (2006) Biochemical analysis of Parkinson's disease-causing variants of Parkin, an E3 ubiquitin-protein ligase with monoubiquitylation capacity. *Hum Mol Genet* **15:** 2059–2075

Hampe C, Ardila-Osorio H, Fournier M, Brice A & Corti O (2006) Biochemical analysis of Parkinson's disease-causing variants of Parkin, an E3 ubiquitin-protein ligase with monoubiquitylation capacity. *Hum Mol Genet* **15:** 2059–2075

Lazarou M, Narendra DP, Jin SM, Tekle E, Banerjee S & Youle RJ (2013) PINK1 drives Parkin self-association and HECT-like E3 activity upstream of mitochondrial binding. *J Cell Biol* **200:** 163–172

Matsuda N, Sato S, Shiba K, Okatsu K, Saisho K, Gautier CA, Sou Y-S, Saiki S, Kawajiri S, Sato F, Kimura M, Komatsu M, Hattori N & Tanaka K (2010) PINK1 stabilized by mitochondrial depolarization recruits Parkin to damaged mitochondria and activates latent Parkin for mitophagy. *J Cell Biol* **189:** 211–221

Narendra DP, Jin SM, Tanaka A, Suen D-F, Gautier CA, Shen J, Cookson MR & Youle RJ (2010) PINK1 is selectively stabilized on impaired mitochondria to activate Parkin. *PLoS Biol* **8:** e1000298

Sriram SR, Li X, Ko HS, Chung KKK, Wong E, Lim KL, Dawson VL & Dawson TM (2005) Familial-associated mutations differentially disrupt the solubility, localization, binding and ubiquitination properties of parkin. *Hum Mol Genet* **14:** 2571–2586

Van Humbeeck C, Waelkens E, Corti O, Brice A & Vandenberghe W (2008) Parkin occurs in a stable, non-covalent, approximately 110-kDa complex in brain. *Eur. J. Neurosci.* **27:** 284–293

Wang C, Tan JMM, Ho MWL, Zaiden N, Wong SH, Chew CLC, Eng PW, Lim TM, Dawson TM & Lim KL (2005) Alterations in the solubility and intracellular localization of parkin by several familial Parkinson's disease-linked point mutations. *Journal of Neurochemistry* **93:** 422–431
